# An internet-based self-help intervention for the reduction of consumption of ultra-processed products and increase of physical activity in Mexican university population: study protocol for a randomized controlled trial

**DOI:** 10.3389/fnut.2024.1325528

**Published:** 2024-07-29

**Authors:** Joel Omar González-Cantero, Leyna Priscila López-Torres, Itzel Refugio Alvarado-Avalos, Fátima López-Alcaraz, Estefania Gasca-Suarez, Adrian Antonio Cisneros-Hernández, Alexandra Valadez, Fabiola Macías-Espinoza, Alejandro Dominguez-Rodriguez

**Affiliations:** ^1^Departamento de Ciencias del Comportamiento, Centro Universitario de los Valles, Universidad de Guadalajara, Ameca, Mexico; ^2^Departamento de Ciencias Sociales, Maestría en Nutrición Humana, Centro Universitario de Ciencias de la Salud, Universidad de Guadalajara, Guadalajara, Mexico; ^3^Maestría en Psicología de la Salud, Centro Universitario de Ciencias de la Salud, Universidad de Guadalajara, Guadalajara, Mexico; ^4^Facultad de Medicina, Universidad de Colima, Colima, Mexico; ^5^Maestría en Nutrición Humana, Centro Universitario de Ciencias de la Salud, Universidad de Guadalajara, Guadalajara, Mexico; ^6^Departamento de Proyectos de Comunicación, Centro Universitario de Arte, Arquitectura y Diseño, Universidad de Guadalajara, Guadalajara, Mexico; ^7^Departamento de Transformaciones Sociales, Centro Universitario de Tlajomulco, Universidad de Guadalajara, Tlajomulco, Mexico; ^8^Departamento de Psicología Aplicada, Centro Universitario de Ciencias de la Salud, Universidad de Guadalajara, Guadalajara, Mexico; ^9^Department of Psychology, Health and Technology, University of Twente, Enschede, Netherlands

**Keywords:** ultra-processed products, ultra-processed foods, physical activity, cognitive behavioral therapy, health psychology, university students, web-based intervention, user experience

## Abstract

**Introduction:**

The consumption of ultra-processed products has been associated with the etiology of various diseases, mainly metabolic diseases. On the other hand, physical activity acts as a protective factor that helps prevent the appearance of this type of disease. In addition to the physical effects, both the consumption of ultra-processed products (UPPs) and sedentary behaviors have been associated with a significant impact on people’s mental health. These problems occur significantly in university students. Online internet interventions are an alternative that has the advantage of reaching a broader sample size and adapting to various problems.

**Methods:**

A randomized controlled clinical superiority trial with two independent groups will be developed with 176 participants. Participants in both groups will be evaluated in 5 steps: (1) pretest, (2) middle of the intervention, (3) post-test, (4) follow-up at 3 months, and (5) follow-up at 6 months. In the experimental group (“UNISALUD”), participants will receive an intervention composed of 11 sessions with interactive elements such as videos, audio, and infographics created through the user experience (UX) principles and based on the health action process approach (HAPA). The participants in the control group will be on the waiting list and will receive treatment 27 days after fulfilling the inclusion criteria. Thus, participants will not receive the treatment immediately.

**Discussion:**

The study is expected to establish the feasibility of a self-help internet-based intervention created based on the user experience methodology and the health action process model, leading to a significant decrease and increase in the consumption of UPPs, ultra-healthy products, and physical activity, respectively.

**Conclusion:**

Internet-based interventions are scarce in Latin America. Due to their potential, this study will provide data about consumption of UPPs, physical activity, and mental health of the Mexican population, which will influence the reduction of health-related complications through prevention strategies or measures.

**Clinical Trial Registration:**ClinicalTrials.gov, NCT05834842.

## 1 Introduction

Ultra-processed products (UPPs) are edible products made mainly or entirely from substances derived from food. It is manufactured through the industrial processes such as hydrogenation (margarine, cookies, and crackers), extrusion, and molding (breakfast cereals, pasta, chips, and gummies), pre-processed for frying (nuggets and French fries) and cannot be made at home (1). The purpose of ultra-processing is to create durable, ready-to-eat, attractive, low-cost, and highly palatable products that often displace the consumption of healthy foods. Most UPPs are rich in energy, sodium, saturated fat, trans fat, and sugar and deficient in vitamins, minerals, and fiber and contain chemical additives (1); hence, they have been identified as low-nutrient foods.

Mexico is the first consumer of UPPs in Latin America (2–4). Approximately 30% of the total calories of the population’s food consumption are sourced from UPPs (4). In the population of the five regions of the country, it is reported that the lockdown forced by the pandemic led to an increase of 23% in the consumption of these products (5). Similarly, 25% of the average monthly expenditure on food is allocated to the purchase, among others, of UPPs, such as beverages, sugars, sweets, snacks, and ready-to-eat products (6). In addition, recently, it has been reported that there is a decrease in the time dedicated to food preparation at home, a condition that could negatively affect the diet and favor the consumption of UPPs (7). Moreover, the regular consumption of UPPs in the adult population has been associated with an increased risk of contracting diseases such as COVID-19 (8) and other pathologies (9) such as increased body weight (10–12), obesity (10), body adiposity (13, 14), neoplasia (15), metabolic syndrome (16), cardiovascular diseases, and diabetes (17–20) and is associated with decreased life expectancy (21).

On the other hand, a sedentary lifestyle or physical inactivity is associated with loss of muscle mass and weight gain (22). In contrast, physical activity (PA) protects against non-communicable diseases such as type 2 diabetes, cardiovascular diseases, and some neoplasia (23). PA levels are determined through metabolic equivalents (METs). The term sedentary lifestyle in the field of sports and exercise is used to describe the absence of PA of moderate to vigorous intensity (24). When it is operationalized, sedentary behavior is defined as “any behavior in the waking state characterized by an expenditure of energy ≤1.5 METs while sitting, reclining, or leaning” (25, p. 5). Examples of sedentary behaviors include watching television, working in an office, driving, or dependence on information and communication technologies (ICT). A sedentary lifestyle or physical inactivity, low levels of PA, and the consumption of UPPs are related to chronic degenerative diseases and premature death (26).

Regarding PA levels, it has been reported in a systematic review that university students spend 7.29 hours a day being sedentary, due to their daily activities, such as studying, doing homework, and attending courses or conferences, among others (27). In another systematic review carried out by López-Valenciano et al. (28), which included 10 studies in which 3,543 university students participated, in 9 of them, PA reductions between 2.9 and 52.8% were observed compared to levels prior to the lockdown for the COVID-19 pandemic; specifically, 5 studies showed a reduction in slight/mild PA (from 32.5 to 365.5%), 3 studies found a decrease in moderate levels (from 14 to 59.7%), 4 studies in moderate to vigorous (3.9 to 56.6%), and 7 studies also showed a reduction in high/vigorous PA (2.9 to 52.8%).

In the Mexican population, it was reported a decrease in PA after 2020, due to the reduction in the time dedicated to transfers for work, school, and leisure purposes; moreover, it has been pointed out an increase in the use of television, computers, and mobile phones, which among others, impacted PA levels (29). In view of the foregoing facts, it is important to emphasize the importance of increasing PA through public policies in Mexico and in consistency with the Objectives of Sustainable Development in the 2030 Agenda established by the United Nations (30). Furthermore, the consumption of UPPs and sedentary behaviors have been associated with a significant impact on people’s mental health, particularly with the increased risk of depression (31–33), anxiety (31, 33, 34), and stress (35). The relationship is bidirectional, as, in addition, the propensity to increase the consumption of UPPs when people present stress has been identified (36, 37).

Nevertheless, eating behavior, a sedentary lifestyle, depressive, anxious, and stress symptoms often have associations with each other (38). For example, a study by Wattick et al. (39) carried out with a university population concluded that food insecurity and the intake of fruits and vegetables are significant predictors of depression in men and women. The same study found that food insecurity and the intake of added sugars were significant predictors of anxiety in both men and women. On the other hand, sedentary behavior in young university students is associated with a greater presence of symptoms of depression (40), anxiety, and stress (40, 41). Therefore, it is important to study the associations between the previously mentioned variables, considering that the mental health of the university population, as reported in a meta-analysis conducted by Wang et al. (42) has a high prevalence of anxiety (29%), depression (37%), and stress (23%). Additionally, it should be noted that according to a report by The United Nations Educational, Scientific, and Cultural Organization (UNESCO), the effects of the pandemic by COVID-19 on university students, although not immediately visible, will be evident in the medium and long term. Therefore, the State and the institutions themselves must generate prevention and intervention measures to mitigate the effects of the pandemic (43).

Thus, to solve the above problems, various interventions have been carried out on university students to improve their eating habits and PA levels. An alternative in this population is Internet interventions, which have the advantage of adapting to various problems.

In the systematic review carried out by Belogianni et al. (44), Internet-based interventions are reported to have a large effect on dietary cognitive outcomes (79% of the participants reported improvement), a moderate effect on dietary intake (35% reported improvement), and weight (57% reported improvement), and a low effect on PA outcomes (both behavioral and cognitive) (20–24% reported improvement). In concrete, self-applied internet interventions can be an option to arrive at a great number of participants (45). In such intervention, the user receives the treatment solely through a web platform or an App. Such interventions are usually composed of videos, text, and audio. Different reviews (46–48) regarding self-administered treatments via the Internet and computer-based treatments are effective in achieving their goals (e.g., decreasing anxiety or depression symptoms). However, one of the main problems is the large dropout rate. To reduce dropout and increase adherence a possibility is to design online interventions based on the needs and characteristics of the user and following the principles of user experience (UX) (49). Although the results of Internet-based interventions are effective for different objectives, these types of interventions are scarce in Latin America, including Mexico (49). More worrisome is the scant research on the impact of consumption of UPPs and its impact on the mental health of the Mexican population.

Therefore, this study aims to evaluate the effectiveness of “UNISALUD,” an Internet-based self-help intervention for reducing consumption of UPPs and increasing PA in the Mexican university population. In addition, as particular objectives we establish:

(a) The impact of UNISALUD on the levels of anxiety, depression, and stress of the participants will be evaluated. (b) From a computer web design perspective, we aim to identify UX regarding the effectiveness of the internet-based self-help intervention.

## 2 Materials and methods

### 2.1 Design

This study will be conducted through a superiority randomized controlled clinical trial (RCT) with two independent groups. It will include intra-subjects at five evaluation moments: (1) pretest, (2) mid-intervention, (3) post-test, (4) follow-up at 3 months, and (5) follow-up at 6 months (45).

**Figure 1:**
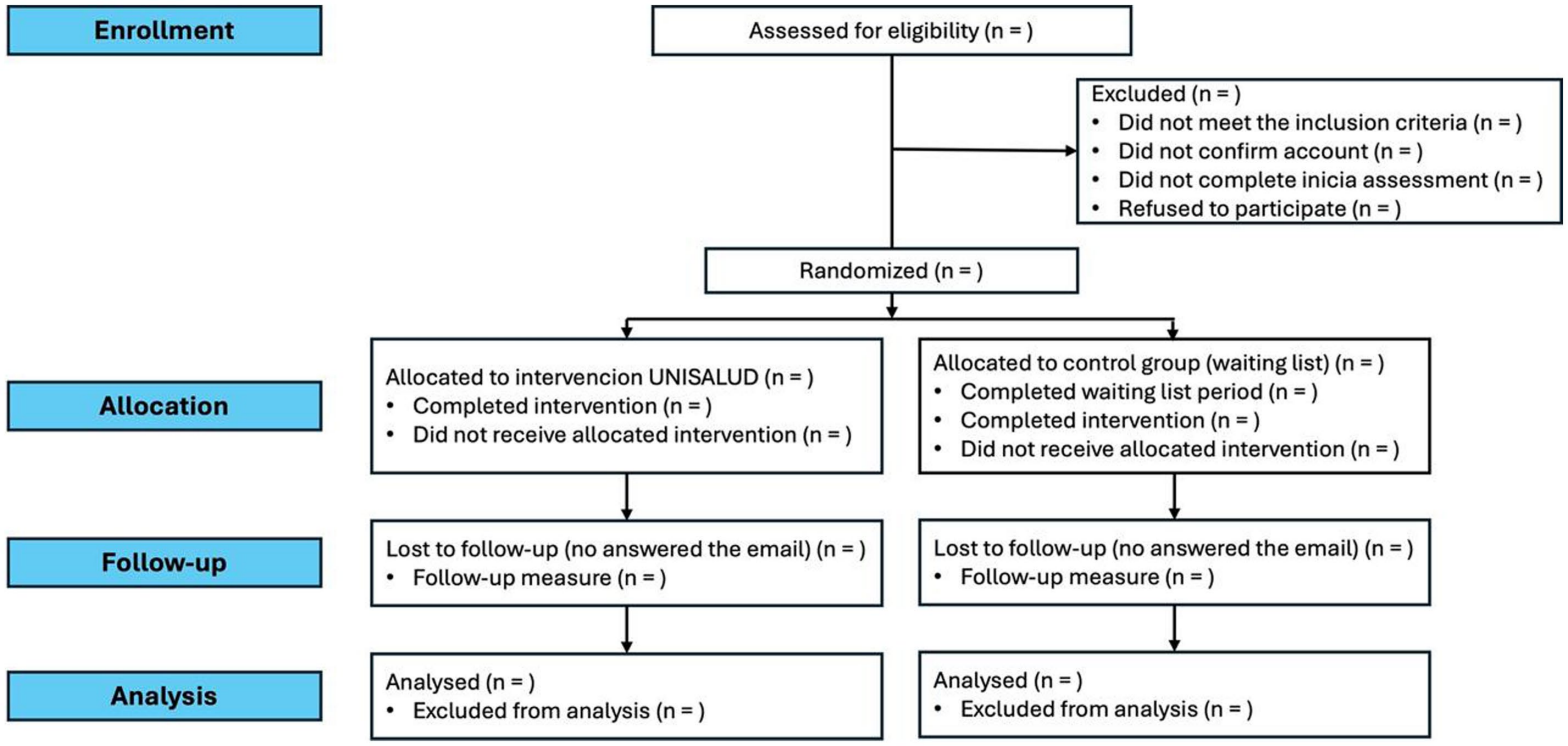
Study design.

Participants will be randomly assigned to one of two groups: the experimental group, “UNISALUD,” composed of 11 sessions and interactive elements such as videos, audio, and infographics; the control group, which will be the waiting list group, the participants in this group will not receive the treatment immediately, it will be measured one time and then a second time 27 days later than the experimental group when it is calculated that the first group has carried out the nine sessions. The post-measures and follow-up will be applied to all the participants to analyze the efficacy of the intervention (Figure 1).

### 2.2 Selection/treatment of subjects

#### 2.2.1 Inclusion and exclusion criteria

##### 2.2.1.1 Inclusion criteria

- Be enrolled in any degree from a university in Mexico.
- Have a device (cell phone, computer, or tablet) with Internet access.
- Be 18 or older.
- Having agreed to participate by giving tacit consent.

##### 2.2.1.2 Exclusion criteria

- Being diagnosed with a psychiatric disorder.
- Being diagnosed with an eating disorder.
- Being under some nutritional (or diet therapeutic) food treatment.
- Have a physical disability or injury that prevents you from engaging in mild-to-moderate physical activity.
- Being under any pharmacological treatment for a medical condition.
- Not completing the initial assessment.

### 2.3 Recruitment

The design used by our work group in previous studies (45) will be replicated:

(1) An invitation will be designed in digital format to inform the population to study about the project. It will be disseminated through digital media and social networks, particularly aimed at groups or pages of young university students and the universities participating in the project.
(2) Participants will enter the platform and need to create an account with an email before starting the evaluation. They will be given informed consent within the platform, which they will need to accept to participate in the study. In this document, the participants will be informed that there is no cost to receive the intervention and that it is aimed at those university students whose lifestyles, related to food and physical activity, are not optimal.

With the registration in the platform, the participants will be informed of the objectives, scope, and development of the intervention.

### 2.4 Randomization process

**Figure 2:**
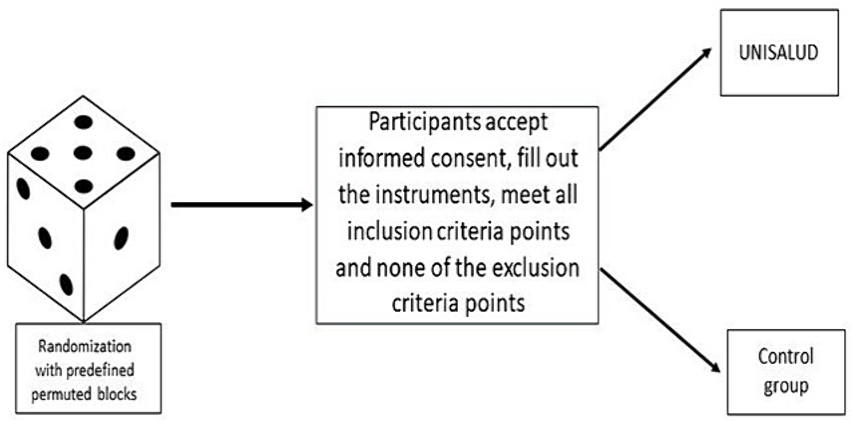
Randomization process.

The randomization procedure will use a permuted blocks algorithm via the Study Randomizer software (50), where a researcher outside the team will obtain the location of the participants with a permuted block algorithm with random block sizes from 6, 9, or 12 allocations before they join the intervention. The process will consist of that once the participant creates an account on the platform, fulfills the inclusion criteria, and does not fulfill any point of the exclusion criteria, will be assigned to the corresponding condition (see Figure 2).

### 2.5 UX methodology to design the intervention

**Figure 3:**
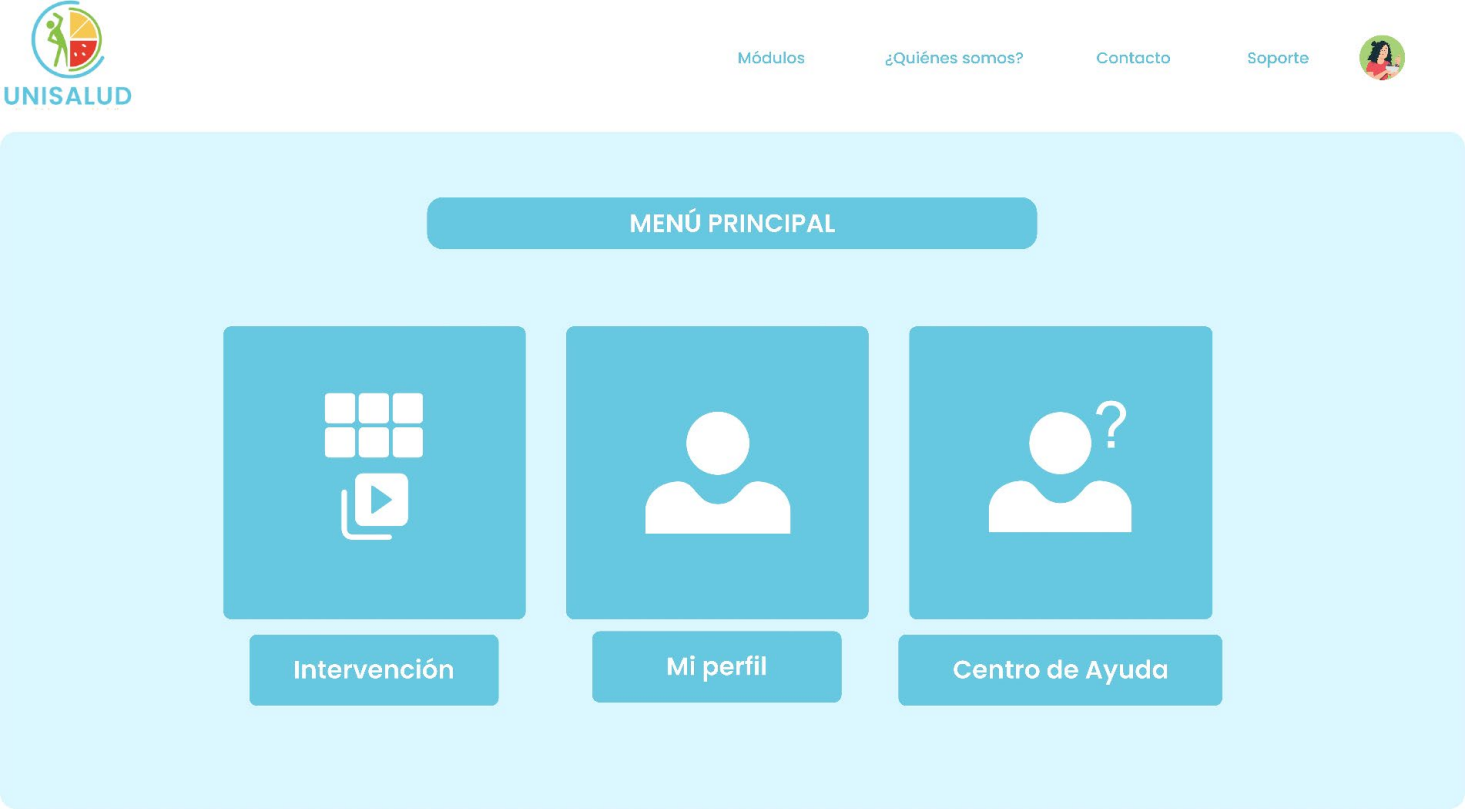
High-fidelity prototype in Figma from the main page.

This intervention was designed through the UX principles (51). This methodology aims to formulate an ideal platform for users, which consists of the following stages:

(I) A survey was conducted with a total of 132 university students; this survey was carried out by 19 students of the Graphic Communication Design Degree of the learning unit Design VII of the University of Guadalajara coordinated by AC-H. Based on the results, the “UNISALUD” identity manual, the logo, and some materials, such as the psychoeducational with the visual collage technique and illustration, were designed by graphic design students. For the videos of behavior modification techniques, a second survey was conducted with 81 students from Chihuahua, San Luis Potosí, Tlaxcala, Jalisco, Puebla, Nayarit and Ciudad de México to find out their preferred video format (presenter vs. avatar), made by 5 bachelor’s degrees in graphic design and communication students.
(II) Semi-structured interviews were conducted with 12 university students from Chihuahua, San Luis Potosí, Tlaxcala, Jalisco, Puebla, Nayarit and Ciudad de México for possible suggestions for an online intervention that provides support for changing eating behavior and physical activity.
(III) Based on the information collected, 20 students from the Bachelor of Design, Art, and Interactive Technology and under the guidance of AC-H and AD-R conducted a search for university student profiles on different university pages from the country.
(IV) With the previous information, affinity mapping was carried out to find similar requests, needs, or suggestions from the participants toward the platform.
(V) User Personas were created.
(VI) Subsequently, User Journey Maps and User Flows were created, along with a proposed site map.
(VII) Wireframes were then drawn with the platform proposal, followed by creating a medium-fidelity prototype in Figma (52).
(VIII) The high-fidelity clickable prototype was designed. In Figure 3, the prototype of the main page is presented.
(IX) Based on the processes, two students from the University Center of Art, Architecture and Design, Mexico, one with a Graphic Communication Design Degree, and another with a Bachelor of Design, Art and Interactive Technologies, all from the University of Guadalajara, they worked with the final interface, which will be delivered to the engineer who will develop the platform. The UX process will be presented in detail in later articles.

### 2.6 Description of the intervention

**Figure 4:**
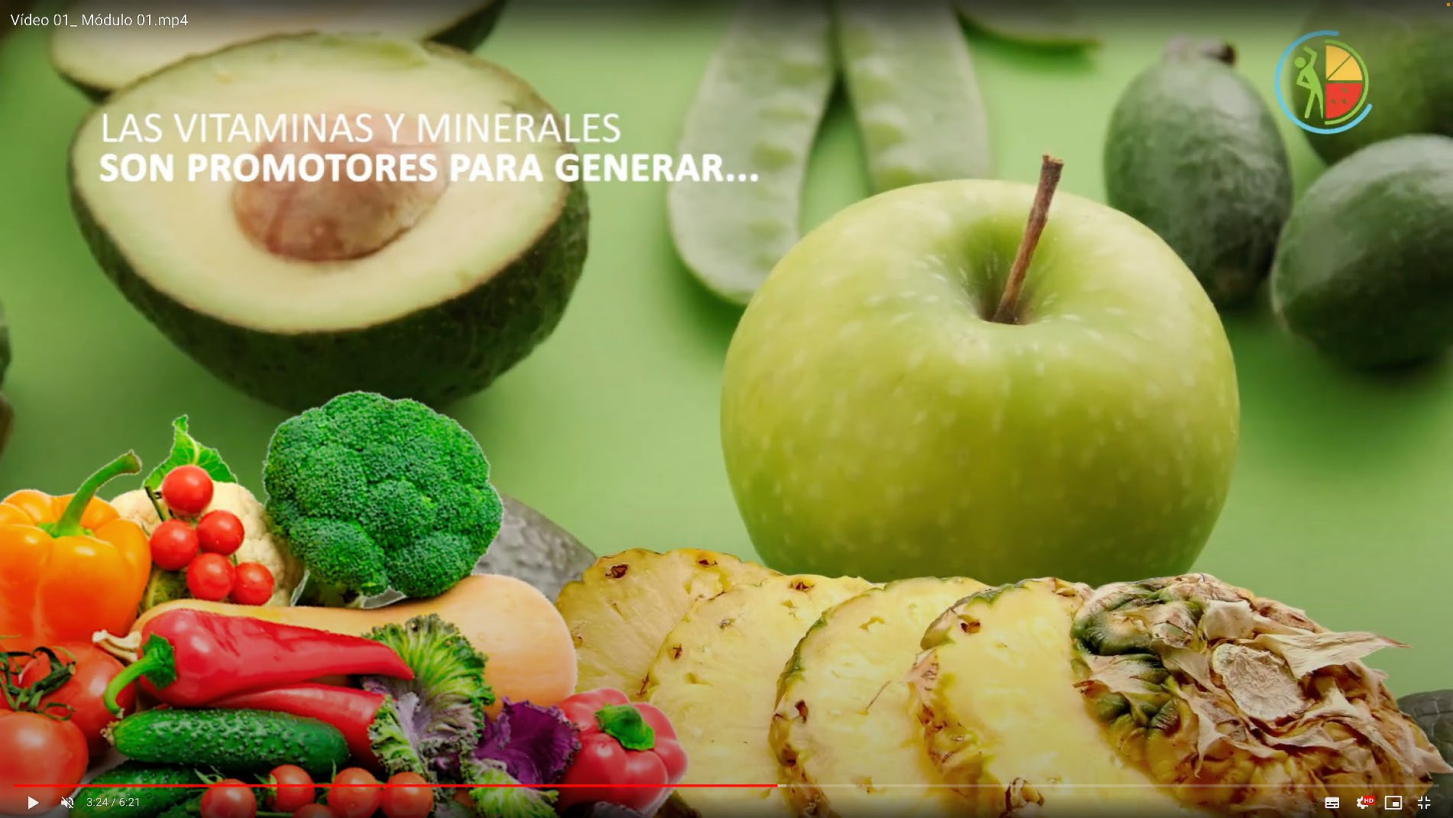
Psychoeducational video Module 1 made by visual collage technique and illustration.

**Figure 5:**
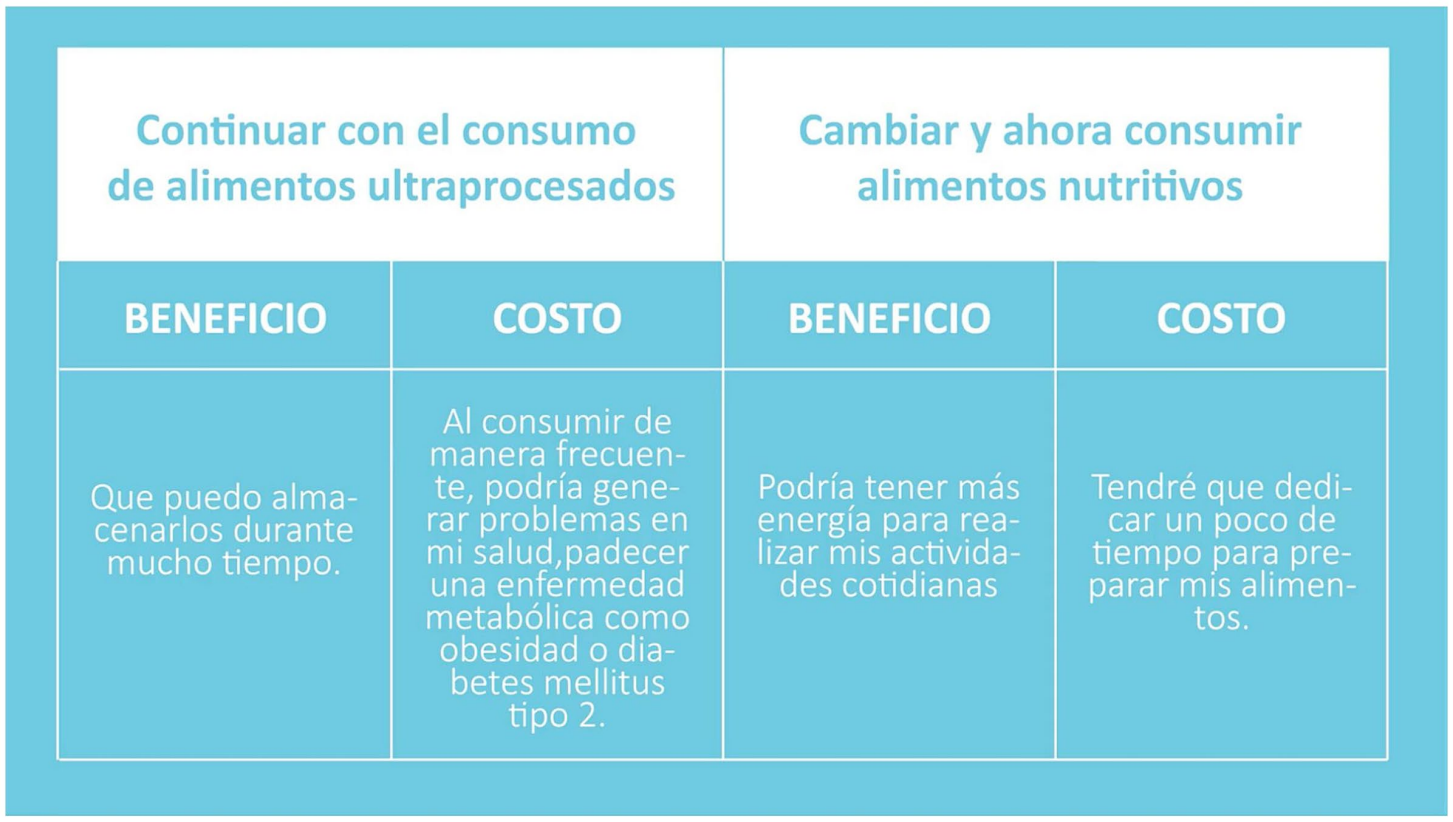
Video Module 1 Decisional balance technique conducted by a presenter.

The “UNISALUD” intervention will be made up of 42 videos, made using the visual collage technique, where illustration, video fragments, and visual interactions are mixed (Figure 4). In some of them, there will be a presenter to achieve captivating visual material to the participant (Figure 5). Similarly, it will be accompanied by digital infographics that will help reinforce the information proposed by the intervention. Before starting the intervention, all the materials will be reviewed by the office of the general lawyer of the University of Guadalajara to (a) register the copyright of the material and (b) guarantee that none of the images used is directly linked to UPPs-registered trademarks.

**Figure 6:**
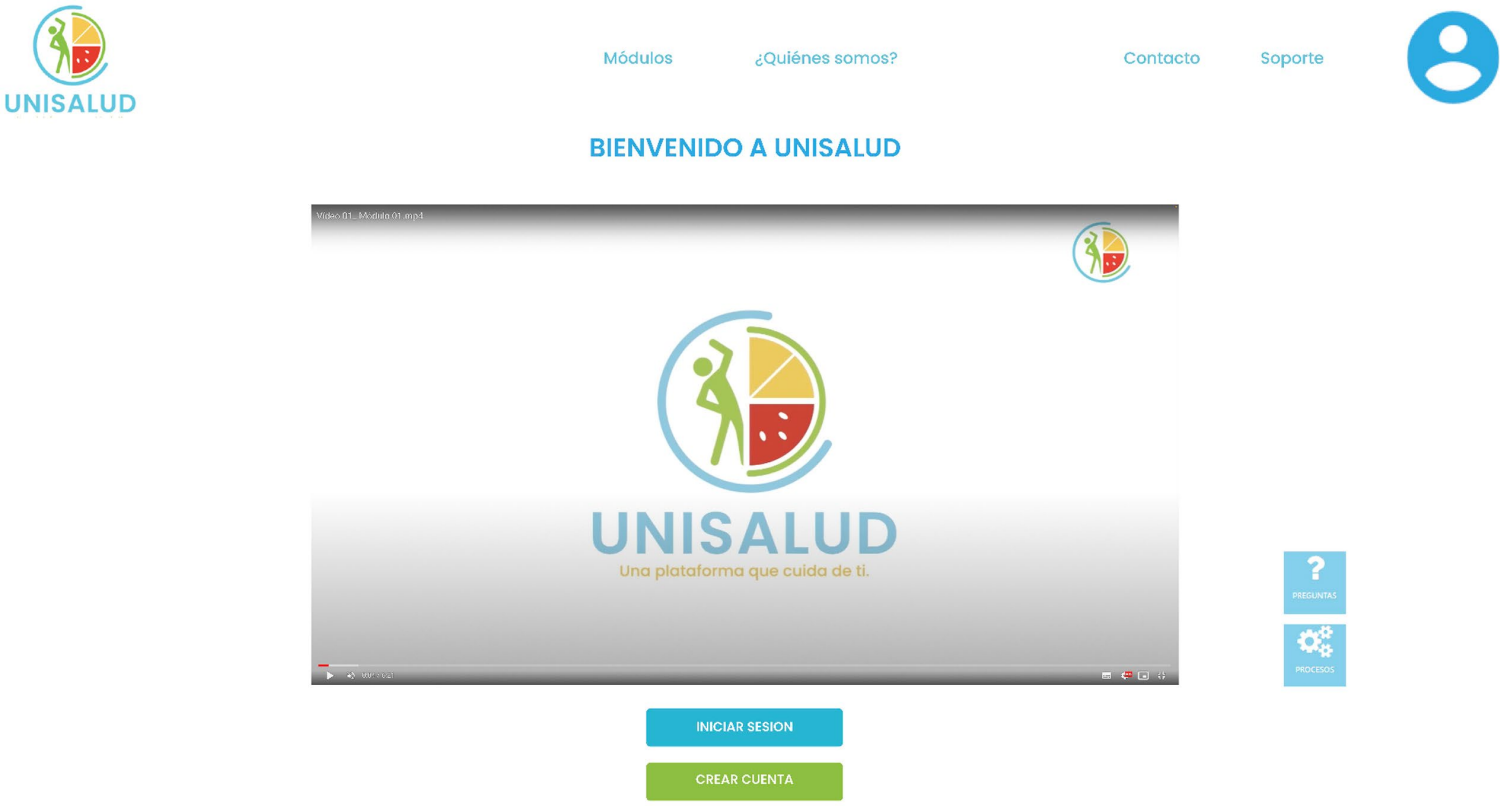
High-fidelity prototype in Figma from the main page.

The previously mentioned material is made up of nine modules (Figure 6), which seek to increase the perception of risk and self-efficacy in relation to the consumption of UPPs and sedentary behaviors presented by university students and how these behaviors are related to the development of symptoms of depression (32), anxiety (31), and stress (36). The intervention is based on the explanatory model of health action process approach (HAPA) (53), and on the Cognitive-Behavioral Therapy (CBT) intervention model. In sessions 10 and 11, which correspond to the 3- and 6-month follow-ups, psychoeducation will be used to analyze the progress and problems raised so that the appropriate techniques can be chosen to solve the problems. The user will be trained in solving problems regarding the reduction of consumption of UPPs and the increase in PA. Table 1 shows the contents and objectives of each of the modules.

**Table 1:** Contents and objectives of the intervention modules.

Module	Content	Objective(s)
1	Benefits of diet and physical activity	Recognize the benefits of adequate nutrition and physical activity
2	Impostor foods and how to get away from them with physical activity	Raise awareness about the health risks of daily consumption of UPPs and identify PA levels and sedentary behaviors
3	What is this I am going to eat? And how do I organize to go move?	Understanding the NOVA classification and identifying and distinguishing processed foods from UPPs. Setting goals and objectives to reduce the consumption of processed foods and engage in PA
4	Science at home to eat fresh	Replace the consumption of UPPs with healthier versions
5	What can I do to move?	Identify replacement strategies for sedentary behaviors
6	How is my diet and physical activity going	Identify the level of compliance with the objectives and the difficulties faced in their achievement
7	The fastest super scoop in the west	Apply planning and organization strategies to reduce the consumption of UPPs
8	How do I train to achieve my goals	Apply planning and organization strategies to increase PA and exercise practice
9	May it not happen to us again	Identify challenges and strategies for establishing behaviors over time
10	Recognizing my achievements	Identify progress and relapses and choose the appropriate techniques to reinforce and/or maintain them (3-month follow-up session)
11	Evaluating my goals	Identify advances and relapses and choose the appropriate techniques to reinforce and/or maintain them (6-month follow-up session)

The HAPA (53) postulates two phases that explain health behaviors: (1) The motivational phase, with motivational processes prior to the behavioral intention and (2) the volitional phase which presents the necessary elements to achieve the behavior after having the intention. In the first phase, it is argued that the perception of risk contributes favorably to the person moving toward contemplation, or in its case, action regarding the behavior they want to change. In addition, in this phase, the expectations of results and self-efficacy favor the person developing the behavioral intention to adopt a difficult behavior. Once the behavioral intention is established, in the second phase, that of volition, it is important to establish specific guidelines on how to develop and maintain the behavior, for which self-regulation skills and strategies are required. Therefore, factors such as planning and self-efficacy are very helpful. Thus, from the CBT, the pertinent techniques were chosen for the approach of each one of the elements of the HAPA, as shown in Table 2.

**Table 2:** Elements of the HAPA and the cognitive-behavioral techniques for its approach.

Phase	Objectives according to each element of the model	Potential mediators	Cognitive-behavioral techniques
Motivation phase	Provide information on the risk of consuming UPPs and physical inactivity	Risk perception	Psychoeducation (54) and decisional balance (55)
	Provide information on the benefits of consuming healthy eating patterns and performing PA	Outcome expectancies	Psychoeducation (54) and decisional balance (55)
	Develop confidence to reduce the consumption of UPPs and increase PA	Action self-efficacy	Goal setting (56) and vicarious experience (57)
	Develop the intention to reduce consumption of UPPs and increase PA	Intention	Behavioral contract (58)
Volition phase	Make plans about consuming minimally processed foods and when, where, how, and with whom to exercise	Action planning	Action planning
	Develop strategies to face the barriers that can interfere with the consumption of minimally processed foods and with the performance of PA	Coping planning	Coping planning (59) and Premack’s principle (60)
	Develop the confidence to maintain or resume consumption of minimally processed foods and PA on a regular basis	Maintenance self-efficacy and recovery self-efficacy	Support cards (57, 61) and problem solving (62)
	Develop strategies to monitor the regular consumption of healthy eating patterns and the performance of PA on a regular basis	Action	Self-monitoring (63)

### 2.7 Study period

The baseline measurement of the participants and therefore the intervention for those that fulfill the inclusion criteria will start in September 2024 and is expected to conclude in March 2025. Within this period, it is expected to include the prospected number of participants. After this period the follow-ups of 3 and 6 months will be included (Table 3).

**Table 3:** Study period.

	Study period						
	Enrollment	Allocation	Post-allocation				
Time point	-t1	0	t1: Pre test	t2: Intervention	t3: Post test	t4: Follow-up 1	t5: Follow-up 2
**Enrollment**							
Eligibility criteria	X						
Informed consent	X						
Allocation		X					
**Interventions**							
Self-applied intervention UNISALUD			X	X	X	X	X
Waiting List group			X		X		
**Assesments**							
Primary outcomes							
Consumption frequency of UPPs			X	X	X	X	X
Physical Activity Questionnaire (IPAQ)			X	X	X	X	X
Self-Efficacy Eating Consumption (SEECS)			X	X	X	X	X
Self-Efficacy Exercise (SEEQ)			X	X	X	X	X
Sedentary Behavior (SBQ-s)			X	X	X	X	X
Health action process approach for consumption of UPPs and sedentary behavior			X	X	X	X	X
**Secondary outcomes**							
Perceived Stress (PSS)			X	X	X	X	X
Anxiety (GAD-7)			X	X	X	X	X
Depressive Symptoms (CES-D)			X	X	X	X	X
System Usability (SUS)					X		

### 2.8 Measures

#### 2.8.1 Primary outcomes

##### 2.8.1.1 Consumption frequency of UPPs

To evaluate and monitor consumption of UPPs, a consumption frequency with emphasis on processing will be used, which was adapted from the NOVA screener for the consumption of ultra-processed foods (64) and the consumption frequency of foods of adolescents and adults (12 years or older), used in the National Health and Nutrition Survey, 2019, which shows the foods usually consumed in Mexico (65). This is a qualitative instrument that evaluates consumption of UPPs in three categories: (a) drinks (12 items); (b) products that replace or accompany meals (26 items); and (c) unhealthy snacks (12 items). Ingestion is reported on a previous day (yes or no) and frequency of consumption per month (never, once a month, once every 15 days, and 1–7 times a week or more than once a day). The cut-off points were delimited based on the studies by Kim et al. (66) and by Costa et al. (64): (a) High consumption: 5 or more products per day, (b) Medium consumption: between 2 and 4 products per day, and (c) low consumption of 1 or fewer products per day.

##### 2.8.1.2 International physical activity questionnaire

The International Physical Activity Questionnaire (IPAQ), validated in the Mexican population (67), is one of the most widely used instruments for measuring the level of physical activity. It is a self-report tool, with its short version comprising seven items where individuals report the duration (in minutes) and frequency (days per week) of engaging in intense activity, moderate activity, walking, or sitting during the last 7 days. Using this information, it is possible to estimate the ranges of metabolic equivalents (METs) by multiplying the time spent on each activity by the number of days per week it was performed. Based on the MET values, the level of physical activity is classified as sedentary behavior (1.0–1.5 METs), low (1.6–2.9 METs), moderate (3.0–5.9 METs), and vigorous (≥ 6 METs).

##### 2.8.1.3 Self-efficacy eating consumption scale

To measure self-efficacy in consumption of ultra-processed products, the SEECS, developed and validated by Palacios et al. (68), will be used. This instrument consists of 21 items with a response option ranging from 1 to 10, where 1 represents the absence of capacity and 10 represents being very capable of reducing the intake of caloric products or sweets and increasing the consumption of healthy foods. This scale shows reliability criteria of (α = 0.93).

##### 2.8.1.4 Self-efficacy exercise questionnaire

To measure self-efficacy in physical activity, the SEEQ developed by Marcus et al. (69) and validated for the Mexican population by Delgado et al. (70) will be used. The SEEQ assesses the degree of confidence people perceive to be physically active. The full scale is composed of five items that assess negative affect, resistance to relapse, and giving oneself time to be physically active. It is Likert-type, and its response options range from 1 (not at all confident) to 5 (extremely confident). The evaluation in the Mexican population has shown reliability criteria (α = 0.81), which indicates good internal consistency.

##### 2.8.1.5 Sedentary behavior questionnaire

The adapted Spanish version of the Latin American population of Sedentary Behavior Questionnaire (SBQ-s) will be used to measure sedentary behaviors (71). The SBQ-s has 11 items that assess the time spent on sedentary behaviors (watching television, eating while sitting, resting while lying down, playing computer or video games while sitting, listening to music while sitting, talking to others or talking on the phone while sitting, doing university work while sitting, reading while sitting, playing a musical instrument, doing crafts, and driving or traveling by car, bus, or subway). These activities are evaluated on both a typical weekday and a weekend day. The response options are: “none,” “15 min or less,” “30 min,” “1 h,” “2 h,” “3 h,” “4 h,” “5 h,” and “more than 6 h.” The time dedicated to each activity is converted into hours. The total hours spent on each activity on a typical weekday and weekend day are summed to obtain the total scores.

##### 2.8.1.6 Health action process approach scale

Renner (72, 73) proposed an evaluation of the different elements of the HAPA model, which are as follows: risk perception, outcome expectations, action self-efficacy, maintenance self-efficacy, recovery self-efficacy, intention, action planning, and coping planning that were created in German and subsequently translated into Spanish (74), which have already been used in a study on eating behavior (75). Based on this proposal, for this study, the HAPA Scale for the evaluation of the consumption of UPPs made up of 21 items and that of sedentary behavior also made up of 21 items were created.

It is considered that there is risk perception with a score of ≥9 and without risk perception with a score of ≤8. To evaluate outcome expectations, they are considered favorable with a score of ≥6 and not favorable with a score ≤5. Regarding the perceived action self-efficacy, a score ≥6 is considered high and a score ≤5 is considered low; the same cut-off points for perceived maintenance self-efficacy and perceived recovery self-efficacy; it is considered that there is favorable behavioral intention with a score of ≥8 and not favorable with a score of ≤7; it is considered that there is favorable action planning with a score of ≥8 and not favorable with a score of ≤7; it is considered that there is favorable coping planning with a score of ≥6 and not favorable with a score of ≤5.

#### 2.8.2 Secondary outcomes

##### 2.8.2.1 Perceived stress scale

The Perceived Stress Scale (PSS) developed by Cohen et al. (76) is used to measure stress. Culturally, it has been adapted in Mexico by González-Ramírez and Landero-Hernández (77). It is a Likert-type of 14 items with response options from 0 (never) to 4 (very often) to evaluate the degree to which situations in one’s life are appraised as stressful. It presents an adequate internal consistency (α = 0.83).

##### 2.8.2.2 Generalized anxiety disorder scale

The GAD-7, developed by Spitzer et al. (78), is used to measure anxiety. Gaitan-Rossi et al. (79) reported a Cronbach’s alpha of 0.93 in a study of the Mexican population. It is a Likert-type of seven items, and its response options range from 0 (not at all) to 3 (nearly every day). A score between 0 and 3 points indicates no perceived anxiety, and a score between 15 and 21 is an indicator of severe perceived anxiety.

##### 2.8.2.3 The Center for Epidemiological Studies Depression Scale

The Center for Epidemiological Studies Depression Scale (CES-D) is a screening scale to detect probable cases of depression. It was designed based on the study of clinical and general populations and is currently one of the most widely used to assess depressive symptomatology in clinical and research settings at international and national levels. This Scale was validated by González-Forteza et al. (80) and consists of 35 questions and contains 5 possible answers ranging from “Scarcely” (0 to 1 day), “Somewhat” (1–2 days), “Occasionally” (3–4 days), “Most” (5–7 days), and “Almost daily” (10–14 days). The scale is validated in the Mexican population (α = 0.90).

#### 2.8.3 Measures of usability and acceptance of the intervention

##### 2.8.3.1 System usability scale

To measure the usability of the platform, the SUS will be used, which is a Likert scale, in which a statement is made, and the respondent indicates the degree of agreement or disagreement with the statement on a scale of 0 to 4, for items 1, 3, 5, 7, and 9, the score contribution is the minor scale position 1. For items 2, 4, 6, 8, and 10, the contribution is five minus the scale position where it is necessary to multiply the sum of the scores by 2.5 to obtain the total value of SU. Scores range from 0 to 100. The scale was validated in the Mexican population. Cronbach’s alpha coefficient for the original version was 0.59 and 0.92 for the positive version, showing that it has good reliability (81).

### 2.9 Possible negative effects and strategies to reduce the risk or damage for the participants

Adopting healthy lifestyles is associated with preventing pathologies such as obesity, hypertension, and diabetes, among others. However, due to the multidimensionality of eating and the complexity of eating behavior, adopting changes in eating can impact various spheres of individuals, for example, affecting social interactions by not consuming UPPs in meetings with other people. In addition, given that the UPPs help reduce the investment of resources (of time and effort) in planning, consumption, and post-consumer activities, reducing the consumption of UPPs will require a greater investment of time for the planning and execution of the actions required to replace its consumption. Similarly, an abrupt elimination of the consumption of UPPs that are habitually found in the diet, linked to affective memories, or consumed under stressful conditions could cause temporary emotional discomfort when they are suppressed from the habitual diet. Due to these conditions, the videos throughout the modules offer alternatives for action in socialization processes in which food and social relationships play an important role, as well as strategies to generate support groups in their process of change. Replacement strategies of UPPs are also presented that allow users to plan activities and save time in planning, purchasing, storing, preparing, and transporting food.

In the case of an increase in PA and a decrease in sedentary behaviors, the gradualness and individuality of the exercises are essential to avoid injuries that may result in the reduction or interruption of physical-sporting activity (82). Accidents can be caused by poor posture when exercising, inappropriate clothing and equipment, excessive activity without a gradual process of adaptation, and lack of warm-up and stretching, among others (83). To prevent and minimize the risk of suffering an injury, the platform will include a series of videos in which a moderator sets an example of how to perform the most common exercises safely and properly.

### 2.10 Data analysis

Descriptive analysis is used to obtain percentages for categorical variables and measures of central tendency and dispersion for continuous variables. A comprehensive analysis will be conducted for each measurement (pretest, mid-intervention, post-test, 3-month follow-up, and 6-month follow-up) for each of the two groups. In accordance with a conservative approach, only valid data from participants who completed the intervention or at least reached the mid-intervention will be utilized for evaluation purposes. However, only participants who have completed all 10 sessions will be considered to have completed the intervention.

To examine whether an internet-based self-help intervention will reduce the consumption of UPPs and increase the frequency of physical activity in a sample of university students along with reducing the symptomatology of anxiety, depression, and stress, a multiple mixed between−/within-subjects ANOVA tests (84) will be conducted (within-group comparisons; Time 1 [T1]—Pretest, time 2 [T2]—mid-intervention, time 3 [T3]—post-test, Time 4 [T4]—3-month follow-up, Time 5 [T5]—6-month follow-up) along with *post-hoc* tests (Tukey HSD), (85) including between-group comparisons with experimental and control groups carried out from Time 1 to Time 5. Only complete questionnaires from T1 to T4 will be considered for statistical analyses.

One-tailed analysis in this experiment means the strength of the effect is expected to be higher between T1 and T3 than between T1 and T5. We expect a stronger effect during the intervention compared to the post-intervention follow-up phase. It also means the experimental group is expected to outperform the control group in terms of a reduction in the symptomatology of anxiety and depression and a decrease in the consumption of UPPs along with the increase in physical activity. The Statistical Package for Social Sciences (SPSS) will be used to conduct the statistical analyses.

### 2.11 Power size calculation

The sample size was calculated using the G*Power 3.1 software. A sample size of 176 individuals was obtained through this program, with the following criteria being considered: a priori power analysis, t-test for two independent means, effect size of 0.5, an error probability of 0.05, a confidence interval of 0.95, and a radius between groups of 1, with the permuted block technique being employed.

The minimum number of participants and power is based on previous interventions delivered virtually and, on a power, analysis calculated *a priori* using the G*Power program (between−/within-group factorial ANOVA 1 - β = 0.95, α = 0.05, Cohen’s *d* = 0.8, which reveals a total sample of 176 participants). A value of 0.80 is considered a large effect size (86). Additionally, it is estimated that 13 participants are added in the total sample to compensate for possible sample mortality (study dropout) for virtually administered interventions with experimental designs that have used long follow-up periods (45).

## 3 Discussion

This study aims to evaluate the efficacy of UNISALUD, an internet-based self-help intervention to reduce the consumption of UPPs and increase the levels of PA and, as a particular objective, to evaluate its effect on the levels of psychopathological symptoms of stress, anxiety, and depression in the Mexican university population. Faced with the challenges of university life, the bidirectional relationship between the consumption of UPPs, physical activity, and mental health, university students could benefit from psychological interventions focused on reducing the consumption of UPPs and increasing PA that have a positive impact on their physical and mental wellbeing.

It is expected that the university population, through the use of an efficient platform in terms of usability, intuition, and accessibility, will identify the risks of consumption of UPPs and sedentary behaviors and, based on psychoeducational videos and cognitive-behavioral techniques, will be able to reduce the consumption of these products and increase their PA levels to positively impact the reduction of psychopathological symptoms of stress, anxiety, and depression. In addition to sensitizing them about the risk if these behaviors are not modified. During the intervention, the participants will learn to identify and differentiate UPPs, as there are studies that have found that this population in Mexico is unaware of their classification (87), which could pose a greater health risk.

Based on the psychoeducational videos and the cognitive-behavioral techniques, it is expected that the consumption of these products will be reduced, strategies to replace these products with healthier and minimally processed alternatives will be implemented, sedentary behaviors will decrease, and PA will increase. If so, this intervention would result in an improvement in the quality of life in this population by reducing risk factors for health, such as the development of chronic non-communicable diseases (10) and/or decreased life expectancy (21), in addition to contributing to the reduction in the symptoms of stress, anxiety, and depression (88–90) possibly derived from the modifications in all areas of daily life and the characteristic changes that derive from entering the university.

Another of the expected benefits is that students can apply strategies to establish long-term behaviors in the face of possible barriers they face and thus maintain healthy behaviors (91, 92). Internet intervention proposals have been shown to potentially transform the healthcare delivery system and empower students to play a more active role in their care while still considering the importance of engagement and the constancy that he shows during sessions (44, 93–95).

Internet interventions have been described in the university population that reduce stress, anxiety, and depression (96–98). In addition to internet interventions that improve PA (99), eating behaviors (91, 100), or both (101–103). In addition, there is evidence of the effectiveness of internet PA interventions with positive effects on depressive symptoms (104). However, interventions aimed at reducing the consumption of UPPs and increasing PA are very limited in Latin America, with certain exceptions (105). To the best knowledge of the authors, this is the first intervention in Latin America that aims to decrease consumption of UPPs and increase PA to benefit the mental health of the participants.

It is important to create internet interventions that are aimed at physical and mental health, which must be adapted to the culture, needs, and context of the target population, offering anonymity, temporal independence, easy accessibility, and scalability (106), otherwise may not be effective as reported by Martínez et al. (107), when implementing an online program for the prevention or early intervention of depression in adolescents, with favorable results for the target population of Chile but not when implementing it in Colombia, both populations with cultural differences, different social contexts and therefore, different psychosocial risks. UNISALUD materials are culturally adapted to the Mexican university population, strengthening the study. A Mexican team of researchers designed the intervention with the participation of university students both within the work team and during the phases described in the methodology. The intervention proposal aims to reinforce the importance of promoting a healthy lifestyle: reduced feeding in UPPs and increased PA in university students. If the results show effectiveness, it can be implemented in other Latin American countries with the respective cultural adaptation, allowing public health authorities to reform future policies on youth health recommendations.

Nonetheless, it is essential to consider adherence, which refers to how people experience or engage with the content of online interventions (108) and poses a significant challenge for this study. It has been reported that dropout rates are higher in self-help interventions (109), because the lack of contact and personal interaction of the online intervention is identified as a disadvantage (110), or this type of intervention manages to be more effective in improving cognitive variables than in modifying behaviors (44). It has even been reported that the reasons for dropping out in this type of intervention are busy hours at the university, decreased motivation, and their belief that the methods used in counseling would not help their improvement (111).

As a main advantage, UNISALUD is the first platform in Latin America to focus on reducing the consumption of UPPs and increasing PA positively impacting mental health. Barquera et al. (112), indicate that interventions should have a person-centered approach that considers a comprehensive perspective with three fundamental pillars: diet, physical activity, and mental health.

Due to the Internet-based modality, some advantages it presents are greater anonymity, comfort, lower cost, and greater flexibility as it allows choosing more appropriate hours so that the student can adapt the program to his/her schedule and pace of work life, as well as greater monitoring capacity, as it will facilitate access with those students who are located in different geographical areas (113). In addition, online interventions are appropriate for university students as this is a population highly familiar with the use of the Internet, who in addition to using the web for academic and recreational purposes, also use it to search for information related to health (114).

The intervention will be completely based on UX, which presents another of the advantages of this work. Ludden et al. (115) mentioned that a better design that is not only intuitive but also pleasant could improve the acceptance and adherence to Internet-based interventions as it could have a positive effect on wellbeing by triggering positive emotions.

Further studies, such as subsequent versions of UNISALUD, could include adaptations of the materials for college students with different disabilities (for example, visual and auditory).

UNISALUD will provide information that could be used to develop Internet interventions for other age groups. This could be relevant, considering that Mexican preschoolers, schoolchildren, and teenagers consume in excess of UPPs (116).

Regarding the limitations, although Internet-based interventions have much potential and the university population is primarily familiar with the web, 39.1% of Mexican university students lack internet access at home. Of those with internet access, 50.1% have slow information transfer or service interruptions (38.6%) (117). This internet access problem is a limitation of the present study, considering that the entire intervention is based on the Internet and that the reproduction of the materials demands a good, stable internet connection. To overcome this limitation, university students can access the intervention via the internet connection provided by all the universities in Mexico, regardless of whether they are private or public.

Another limitation is that we will not be able to assess the health status of the participants, which comprises multiple aspects that may be related to adherence to treatment and, thus, to the results of the intervention. In a further version of the intervention, we could request the support of state governments and universities to help assess the students’ health and afterward provide the intervention to control for this variable and the impact that could have on the results.

A last limitation is related to the self-reported instrument, where the responses would be more reliable if administered by trained personnel. However, the limited resources in this study do not allow this. To solve this, future studies could include a sub-sample assessed by trained personnel and compare the results of the participants who answered in a self-applied format to explore whether there are differences in the answers.

## 4 Conclusion

In summary, our intervention, UNISALUD, is among the first online interventions aimed at reducing UPP consumption and increasing physical activity performance and mental health in university students in Mexican Universities. The study results can help provide a further intervention aimed at the general population in Mexico.

## Ethics statement

This study was approved and ruled with number CI-03722 by the Research Ethics Committees (registry CONBIOETICA-14_CEI-002-20191003), Research (registry 22 CI 14039014), and Biosafety (22 CB 14039015), of the University Center of Health Sciences of the University of Guadalajara, Mexico. It is registered in ClinicalTrials.gov, Identifier: NCT05834842. Participants will provide their informed consent to participate in this study. All the information obtained in this study will be handled anonymously. In any case, each participant will be assigned a code as a registry, where all the comparative data will be processed anonymously, limiting access to the database only to personnel linked to the development of the study.

## Author contributions

JG-C: Investigation, Methodology, Project administration, Resources, Supervision, Writing – original draft, Writing – review & editing, Funding acquisition. LL-T: Funding acquisition, Investigation, Methodology, Project administration, Resources, Supervision, Writing – original draft, Writing – review & editing. IA-A: Investigation, Project administration, Resources, Supervision, Writing – original draft, Writing – review & editing. FL-A: Investigation, Project administration, Resources, Writing – original draft, Writing – review & editing, Methodology. EG-S: Investigation, Methodology, Project administration, Resources, Writing – original draft, Writing – review & editing. AC-H: Investigation, Methodology, Project administration, Resources, Writing – original draft, Writing – review & editing, Software, Supervision. AV: Investigation, Methodology, Resources, Supervision, Writing – original draft, Writing – review & editing. FM-E: Investigation, Methodology, Resources, Supervision, Writing – original draft, Writing – review & editing, Funding acquisition. AD-R: Investigation, Methodology, Resources, Supervision, Writing – original draft, Writing – review & editing, Conceptualization, Project administration, Software.
